# Associations of Lifestyle Intervention Effect with Blood Pressure and Physical Activity among Community-Dwelling Older Americans with Hypertension in Southern California

**DOI:** 10.3390/ijerph17165673

**Published:** 2020-08-05

**Authors:** Mei-Lan Chen, Jie Hu, Thomas P. McCoy, Susan Letvak, Luba Ivanov

**Affiliations:** 1 Byrdine F. Lewis College of Nursing and Health Professions, Georgia State University, Atlanta, GA 30303, USA; 2 Gerontology Institute, Georgia State University, Atlanta, GA 30303, USA; 3 College of Nursing, The Ohio State University, Columbus, OH 43210, USA; hu.1348@osu.edu; 4 School of Nursing, University of North Carolina at Greensboro, Greensboro, NC 27402, USA; tpmccoy@uncg.edu (T.P.M.); saletvak@uncg.edu (S.L.); 5 College of Nursing, Chamberlain University, Downers Grove, IL 60515, USA; livanov@chamberlain.edu

**Keywords:** older adults, high blood pressure, lifestyle intervention, physical activity, hierarchical multiple regression analyses, social support, perceived stress

## Abstract

A healthy lifestyle and regular physical activity are highly recommended for older adults. However, there has been limited research into testing lifestyle intervention effects on physical activity in older adults with hypertension. The purpose of this study was to assess the association of lifestyle intervention effects with physical activity and blood pressure in older adults with hypertension, accounting for social support and perceived stress as control variables. This study performed a secondary analysis of a two-arm randomized controlled trial. A total of 196 participants were randomly assigned to a six-month lifestyle intervention group or a control group. Hierarchical multiple regression analyses demonstrated that lifestyle intervention effects were not significantly associated with improvements in physical activity and blood pressure, but the final regression models were statistically significant (all *p* < 0.001). The result revealed that only physical activity frequency at baseline was significantly related to improvement in physical activity. Systolic blood pressure (SBP) at baseline and monthly income were significantly associated with change in SBP, while age and diastolic blood pressure (DBP) at baseline were significantly related to change in DBP. The findings provide empirical evidence for developing and optimizing lifestyle interventions for future research and clinical practice in this population.

## 1. Introduction

Hypertension, a significant cause of morbidity and mortality, is one of the leading risk factors for stroke and cardiovascular diseases. Nearly 46% of adults, an estimated 116.4 million adults, have high blood pressure in the United States. However, the hypertension control rate is only about 25% among adults with hypertension [1]. The death rate resulting from high blood pressure increased 25.7% from 2007 to 2017 [1,2]. The estimated annual average direct and indirect cost of hypertension is $55.9 billion; by 2035, the estimated direct costs of hypertension could increase to $220.9 billion [1].

High prevalence and poor control of hypertension remain significant burdens for older Americans. Prevalence of high blood pressure is 59.2% in those 45–64 years of age and 78.2% in those ≥65 years of age [1]. Approximately 72% of older adults self-reported that they were taking antihypertensive medication, but only 30% of older adults with hypertension had controlled hypertension [1,3]. Older women have a higher prevalence of hypertension and a higher rate of uncontrolled hypertension than older men. Nearly 65% of older men have hypertension and 47% reported uncontrolled hypertension, compared to 73% of older women with hypertension and 54% with uncontrolled hypertension [4].

It is known that high blood pressure can be controlled if effective interventions are undertaken [1,5]. However, hypertension is poorly controlled by many older Americans. Because many older Americans have uncontrolled high blood pressure, it is important to promote effective interventions to better control hypertension in older adults, especially for older women. Lifestyle interventions are highly recommended for older adults, including regular physical activity, a healthy dietary pattern, limited alcohol intake, and healthy weight [5,6]. However, there has been limited research into testing lifestyle intervention effects on physical activity and blood pressure in older adults with hypertension over time. In addition, stress and social support may influence engagement in regular physical activity and active lifestyles in older adults. Studies have indicated that stress levels are negatively associated with physical activity levels [7,8]. In addition, social support may predict physical activity and healthy lifestyle initiation and maintenance [9,10,11]. Accordingly, the purpose of this study was to examine the association of lifestyle intervention effect with blood pressure and physical activity in older adults with hypertension, accounting for social support and perceived stress as control variables.

The conceptual framework guiding the study has been described in our previous study ([12], p. 2). Briefly, the current study assumes that there is an association between person (perceived stress), environment (social support), and the outcomes (blood pressure, physical activity). We hypothesized that the lifestyle intervention effect is significantly related to changes in blood pressure and physical activity, accounting for socio-demographic factors, social support, and perceived stress.

## 2. Materials and Methods

### 2.1. Data Source and Study Design

This study used a secondary dataset from the Well Elderly 2 Study. The data of the Well Elderly 2 Study in this study were provided by the Inter-University Consortium for Political and Social Research (ICPSR) [13]. The research design, methods, and the intervention of the Well Elderly 2 Study have already been reported elsewhere by Clark and colleagues [13,14,15,16]. In brief, in the Well Elderly 2 Study, a total of 460 independent-living older adults aged 60–95 were recruited from community-based sites in the greater Los Angeles metropolitan area, in California [13,15,16]. All participants were randomly assigned to either the intervention group or the control group. During the first six months, the intervention group received a lifestyle intervention while the control group did not receive the intervention. The modules of the 6-month lifestyle intervention consisted of “Introduction to the Power of Activity; Aging, Health, and Activity; Transportation; Safety; Social Relationships; Cultural Awareness; Finances; and Integrative Summary: Lifestyle Redesign Journal” ([17], p. 355). The format of the intervention comprised monthly community outings, 2-h group meetings per week, and up to ten, one-on-one, 1-h sessions [14,15,17].

The current secondary analysis investigated only data collected during the first six months of the original study. Subjects who self-reported taking antihypertensive medication were selected as the study sample.

### 2.2. Measurements

#### 2.2.1. Socio-Demographic Characteristics, Social Support, and Perceived Stress

Details of the measures of socio-demographic characteristics, perceived stress, and social support were reported in our previous study ([12], p. 2). Briefly, socio-demographic characteristics included age, sex, ethnicity, monthly income, and educational level. The Lubben Social Network Scale (LSNS) was used to measure social support. Scores range from 0 to 50, where higher scores suggest higher levels of social support [12,18,19,20,21]. The adapted Perceived Stress Scale (PSS), an 18-item scale, was used to measure perceived stress [12]. Scores of the adapted PSS range from 18 to 90, where higher scores suggest higher levels of perceived stress [22,23]. In the current study, Cronbach’s alpha coefficients of the LSNS and the adapted PSS were 0.75 and 0.85, respectively.

#### 2.2.2. Blood Pressure

Each participant’s blood pressure (systolic blood pressure and diastolic blood pressure) was measured when they were in a sitting position. Two blood pressure measurements were obtained and the mean of the blood pressure readings was calculated and used for analysis [13].

#### 2.2.3. Physical Activity

In the Well Elderly 2 study, Meaningful Activity Participation Assessment-Frequency (MAPA-F), a 29-item tool, was used to examine activity participation frequency for 29 diverse activities including physical activity [24]. The MAPA-F is a reliable and valid tool to measure meaningful activities, such as physical activity in older adults [24,25]. All items for each activity using Likert scaling are scored from 0 (not at all) to 6 (every day). The possible scores of the MAPA-F range from 0 to 174; higher scores indicate greater levels of meaningful activity participation. In the current study, physical activity was measured with physical activity frequency, using the MAPA-F. The possible scores of physical activity frequency range from 0 to 6; higher scores indicate higher levels of physical activity frequency. In this study, Cronbach’s alpha coefficient of the MAPA-F was 0.82.

### 2.3. Ethical Approval

The present study was approved by the Institutional Review Board (IRB) of the University of North Carolina at Greensboro (approval number: 14-0428).

### 2.4. Statistical Analysis

The primary goal of the statistical analysis in the current study was to test the associations of lifestyle intervention effect with changes in blood pressure and physical activity, from pre-test (baseline) to post-test (6 months), accounting for social support and perceived stress as control variables. Descriptive statistics and hierarchical multiple regression (HMR) were used to analyze the sample characteristics and make predictions on criterion variables [12,26,27]. The change in blood pressure was modeled using independent variables including socio-demographic variables (age, sex, ethnicity, education, monthly income), blood pressure at baseline, lifestyle intervention effect (intervention vs. control), social support (LSNS score) at baseline, change in LSNS score, perceived stress (PSS score) at baseline, and change in PSS score through HMR analysis. For modeling the change in physical activity frequency, socio-demographic variables, physical activity frequency at baseline, lifestyle intervention effect, LSNS score at baseline, change in LSNS score, PSS score at baseline, and change in PSS score as independent variables were entered. Data were analyzed using SPSS software (version 23; IBM corp., Armonk, IL, USA). A two-tailed *p*-value <0.05 was considered statistically significant.

## 3. Results

### 3.1. Characteristics of the Sample

A total of 196 participants (mean age 74.8 years ± 7.7) were included in the analyses of this study. The socio-demographic characteristics of the sample were detailed in our previous study ([12], pp. 3–4). In brief, the majority of participants were female (63%). Most self-identified as African American (40%) or White (33%). Ninety-one participants (46%) had received more than a high school education; one hundred and four participants (54%) had a monthly income lower than $1000. At baseline, the two study groups had no significant differences in socio-demographic characteristics (all *p* ≥ 0.05) ([12], p. 3). Table 1 indicates the distribution of means for the continuous variables for the two groups. In the intervention group, the mean systolic blood pressure (SBP), mean diastolic blood pressure (DBP), and the average physical activity frequency score were 141.9 ± 18.7 mmHg, 77.1 ± 12.5 mmHg, and 3.9 ± 2.2, respectively. In the control group, the mean SBP, mean DBP, and the average physical activity frequency score were 146.6 ± 21.6 mmHg, 78.9 ± 11.2 mmHg, and 3.9 ± 2.2, respectively. At baseline, there were no significant differences between the two study groups based on these study variables (all *p* ≥ 0.05).

### 3.2. Association of Lifestyle Intervention Effect with Changes in Blood Pressure

Table 2 indicates the associations with the independent variables at each step for predicting changes in systolic blood pressure (SBP; post-baseline) in a final hierarchical regression model, accounting for lifestyle intervention effect, social support, and perceived stress. As shown in Table 2, there was no significant relationship between the lifestyle intervention effect and change in SBP, but the final regression model was statistically significant (R^2^ = 0.36; *p* < 0.001). In this model, monthly income ($1000–$1999 vs. <$1000) was significantly related to change in SBP (β = −0.21; *p* < 0.05), and SBP at baseline was significantly associated with change in SBP (β = −0.45; *p* < 0.001), adjusting for all other factors.

Table 3 shows the final hierarchical regression model for the associations with the independent variables in predicting change in diastolic blood pressure (DBP; post-baseline), accounting for lifestyle intervention effect, social support, and perceived stress. The results indicate that the lifestyle intervention effect was not significant associated with change in DBP, but the final regression model was statistically significant (R^2^ = 0.49; *p* < 0.001). In the final model, age was significantly associated with change in DBP (β = −0.17; *p* < 0.05); DBP at baseline was significantly related to change in DBP (β = −0.56; *p* < 0.001), adjusting for all other factors.

### 3.3. Association of Lifestyle Intervention Effect with Changes in Physical Activity Frequency

Table 4 presents the associations with the independent variables at each step for predicting change in physical activity frequency (post-baseline), accounting for lifestyle intervention effect, social support, and perceived stress. As shown in Table 4, the lifestyle intervention effect was not significantly related to the change in physical activity frequency, but the final regression model was statistically significant (R^2^ = 0.33; *p* < 0.001). In the final model, physical activity frequency at baseline was significantly associated with change in physical activity frequency (β = −0.57; *p* < 0.001), adjusting for all other factors.

## 4. Discussion

This study tested the associations of lifestyle intervention effect with blood pressure and physical activity in older adults with hypertension. The results of this secondary analysis revealed that there were no statistically significant lifestyle intervention effects on blood pressure and physical activity, but the final regression models were statistically significant, indicating at least some other model effects were significant. The findings of the hierarchical multiple regression analysis indicated that SBP at baseline, and monthly income ($1000–$1999 vs. <$1000), were significant predictors for predicting change in SBP. Age and DBP at baseline were significant predictors for predicting change in DBP. Physical activity frequency at baseline was the only significant predictor for predicting change in physical activity frequency.

The results of the current analysis are inconsistent with previous studies. Hageman et al. (2014) examined a community-based clinical trial to test the effects of lifestyle interventions in midlife and older women with prehypertension [28]. Participants were randomly assigned to three groups: a standard advice group (control group), a web-based lifestyle intervention group, and a print-mailed lifestyle intervention group. The results revealed that the web-based intervention group had significantly greater reduction in systolic blood pressure when compared with the control group.

Similarly, Fernandez et al. (2008) tested the effect of a center–based lifestyle intervention on systolic blood pressure in minority older individuals with hypertension [29]. The study found statistically significant reductions on systolic blood pressure in the intervention group. Lin and colleagues (2014) examined a 12-month, community-based lifestyle intervention in middle-aged and older adults [30]. The result showed that there were significant reductions in systolic blood pressure and diastolic blood pressure in the intervention group. Hence, lifestyle changes play an important role in management of hypertension in older adults.

In the current study, we found that lifestyle intervention effects were not significantly related to improvements in physical activity and blood pressure. One potential explanation for the lack of intervention effects is that the dosing of the intervention, the 6-month duration of the original study, was insufficient for changes in blood pressure and physical activity. In addition, the lifestyle intervention emphasized the impact of daily activity on health ([15], p. 783), but the format of the intervention did not include structured physical activity sessions. Possibly, it did not have substantial intervention effects due to a lack of direct intervention related to physical activity.

Regular physical activity is an effective way to prevent and control hypertension in older adults [2]. Studies have revealed that aerobic exercise significantly improves resting blood pressure in hypertensive older adults [31,32,33]. Previous studies highly recommend that physical activity interventions should be included in lifestyle programs for older adults, but older adults in the United States engage in less physical activity than young adults [2,34,35,36]. Studies found that the enjoyment of physical activity, cultural health beliefs, encouragement of family and friends, and beliefs on physical activity benefits can impact older adults’ physical activity engagement [37,38,39,40]. Sex, ethnicity, educational level, and economic difficulties also appear to affect regular physical activity participation among older adults [33,37]. Hence, a better understanding of racial and social disparities, and the psychological processes involved in initiating and maintaining regular physical activity is necessary for older adults with hypertension [41,42].

This study had the following limitations. First, the Meaningful Activity Participation Assessment-Frequency (MAPA-F) was used to measure physical activity frequency, but the duration and intensity of physical activity were not available in the MAPA-F. Second, the Well Elderly 2 Study did not conduct a readiness-to-change screening [15]. Lack of the readiness-to-change screening can impact the effects of the intervention. Lastly, the participants in this study were nonclinical urban community dwellers [12,17]. This may have limited the generalizability of the findings.

## 5. Conclusions

Our findings in the study make an important contribution to the literature on lifestyle intervention and physical activity among older adults with hypertension. Baseline blood pressure, income, and physical activity frequency were important predicting factors contributing to the changes in blood pressure and physical activity frequency in this sample. Future research on lifestyle intervention and physical activity among older adults with hypertension should consider including increased doses of the intervention, structured physical activity sessions, and the goal setting for physical activity that is recommended for older adults. As the older population is growing significantly and many older adults have hypertension, lifestyle interventions in combination with physical activity interventions are strongly recommended for future studies and clinical practice.

## Figures and Tables

**Table 1:** Distribution of means for continuous variables by study group (*N* = 196).

Variable	Intervention Group	Control Group	*p*
	(*n* = 103)	(*n* = 93)	
Age (years), mean ± *SD*	74.2 ± 7.7	75.3 ± 7.7	0.304
Perceived stress (PSS)	43.8 ± 11.0	43.6 ± 10.5	0.885
Social support (LSNS)	27.1 ± 8.5	27.3 ± 9.7	0.907
Systolic blood pressure (mmHg)	141.9 ± 18.7	146.6 ± 21.6	0.131
Diastolic blood pressure (mmHg)	77.1 ± 12.5	78.9 ± 11.2	0.328
Physical activity frequency (0–6 score)	3.9 ± 2.2	3.9 ± 2.2	0.899

*Note*: *SD* = Standard Deviation; PSS = Perceived Stress Scale; LSNS = Lubben Social Network Scale.

**Table 2:** Hierarchical multiple regression analyses examining the association of the lifestyle intervention effect with change in systolic blood pressure (*n* = 118).

Predictor Variable	Change in Systolic Blood Pressure	
	∆*R*^2^	β ^a^ (95% CI)
Step 1: Socio-demographic variables	0.16	
Age (years)		−0.07 (−0.69, 0.29)
Sex		
Men ^b^		
Women		0.11 (−3.07, 12.29)
Ethnicity		
White ^b^		
African American		−0.12 (−14.82, 4.23)
Hispanic/Latino		−0.05 (−16.33, 10.52)
Asian		0.03 (−17.59, 24.56)
Other		−0.10 (−27.37, 8.32)
Education		
Lower than high school education ^b^		
High school diploma		−0.12 (−17.58, 5.43)
Some college/technical school degree		0.03 (−10.27, 12.87)
Four years of college or higher		−0.01 (−16.42, 15.11)
Monthly Income		
<$1000 ^b^		
$1000–$1999		−0.21 * (−20.75, −1.56)
$2000–$2999		−0.03 (−13.34, 9.56)
*≥*$3000		−0.01 (−12.40, 10.87)
Step 2	0.16 ***	
Systolic blood pressure at baseline (mmHg)		−0.45 *** (−0.67, −0.28)
Step 3: Lifestyle intervention effect	<0.01	
Control group ^b^		
Intervention group		0.05 (−4.84, 9.32)
Step 4	0.04	
LSNS score at baseline (points)		0.15 (−0.10, 0.74)
Change in LSNS score		0.12 (−0.24, 1.06)
PSS score at baseline (points)		0.13 (−0.16, 0.72)
Change in PSS score		−0.04 (−0.52, 0.36)
Total *R*^2^	0.36 ***	

*Note*: ^a^ β shown is for the last step; ^b^ Reference category; CI = confidence interval; LSNS = Lubben Social Network Scale; PSS = Perceived Stress Scale; * *p* < 0.05, *** *p* < 0.001 (two-sided).

**Table 3:** Hierarchical multiple regression analyses examining the association of the lifestyle intervention effect with change in diastolic blood pressure (*n* = 117).

Predictor Variable	Change in Diastolic Blood Pressure	
	∆*R*^2^	β ^a^ (95% CI)
Step 1: Socio-demographic variables	0.21 *	
Age (years)		−0.17 * (−0.63, −0.03)
Sex		
Men ^b^		
Women		0.12 (−1.36, 8.22)
Ethnicity		
White ^b^		
African American		−0.05 (−7.05, 4.31)
Hispanic/Latino		−0.04 (−9.30, 6.32)
Asian		0.07 (−6.91, 18.40)
Other		−0.03 (−12.44, 9.22)
Education		
Lower than high school education ^b^		
High school diploma		−0.06 (−8.44, 4.72)
Some college/technical school degree		−0.19 (−12.30, 1.05)
Four years of college or higher		−0.03 (−10.60, 8.16)
Monthly Income		
<$1000 ^b^		
$1000–$1999		−0.08 (−8.68, 2.90)
$2000–$2999		0.08 (−3.73, 10.04)
*≥*$3000		−0.07 (−9.87, 4.08)
Step 2	0.26 ***	
Diastolic blood pressure at baseline (mmHg)		−0.56 *** (−0.85, −0.47)
Step 3: Lifestyle Intervention Effect	0.01	
Control group ^b^		
Intervention group		0.12 (−0.75, 7.76)
Step 4	0.01	
LSNS score at baseline (points)		−0.02 (−0.28, 0.22)
Change in LSNS score		0.03 (−0.32, 0.46)
PSS score at baseline (points)		−0.02 (−0.29, 0.24)
Change in PSS score		−0.08 (−0.38, 0.15)
Total *R*^2^	0.49 ***	

*Note*: ^a^ β shown is for the last step; ^b^ Reference category; CI = confidence interval; LSNS = Lubben Social Network Scale; PSS = Perceived Stress Scale; * *p* < 0.05, *** *p* < 0.001 (two-sided).

**Table 4:** Hierarchical multiple regression analyses examining the association of the lifestyle intervention effect with change in physical activity frequency (*n* = 167).

Predictor Variable	Change in Physical Activity Frequency	
	∆*R*^2^	β ^a^ (95% CI)
Step 1: Socio-demographic variables	0.01	
Age (years)		−0.02 (−0.04, 0.03)
Sex		
Men ^b^		
Women		0.03 (−0.49, 0.70)
Ethnicity		
White ^b^		
African American		−0.04 (−0.85, 0.57)
Hispanic/Latino		−0.003 (−0.96, 0.93)
Asian		0.04 (−1.17, 2.15)
Other		0.02 (−1.07, 1.41)
Education		
Lower than high school education ^b^		
High school diploma		−0.06 (−1.09, 0.52)
Some college/technical school degree		−0.05 (−1.02, 0.61)
Four years of college or higher		−0.02 (−1.33, 1.06)
Monthly Income		
<$1000 ^b^		
$1000–$1999		0.06 (−0.42, 1.03)
$2000–$2999		0.11 (−0.27, 1.54)
*≥*$3000		0.11 (−0.21, 1.58)
Step 2	0.29 ***	
Physical activity frequency at baseline		−0.57 *** (−0.65, −0.39)
Step 3: Lifestyle intervention effect	<0.01	
Control group ^b^		
Intervention group		0.02 (−0.45, 0.63)
Step 4	0.02	
LSNS score at baseline (points)		−0.05 (−0.04, 0.02)
Change in LSNS score		−0.01 (−0.05, 0.04)
PSS score at baseline (points)		−0.12 (−0.05, 0.00)
Change in PSS score		−0.16 (−0.06, 0.00).
Total *R*^2^	0.33 ***	

*Note*: ^a^ β shown is for the last step; ^b^ Reference category; CI = confidence interval; LSNS = Lubben Social Network Scale; PSS = Perceived Stress Scale; *** *p* < 0.001 (two-sided).
